# Detection of SARS‐CoV‐2 in saliva and characterization of oral symptoms in COVID‐19 patients

**DOI:** 10.1111/cpr.12923

**Published:** 2020-10-19

**Authors:** Lili Chen, Jiajia Zhao, Jinfeng Peng, Xiaoshuang Li, Xuliang Deng, Zhi Geng, Zhenyu Shen, Fengyuan Guo, Qianwen Zhang, Yang Jin, Lin Wang, Songlin Wang

**Affiliations:** ^1^ Department of Stomatology Union Hospital Tongji Medical College Huazhong University of Science and Technology Wuhan China; ^2^ Hubei Province Key Laboratory of Oral and Maxillofacial Development and Regeneration Wuhan China; ^3^ Department of Geriatric Dentistry Peking University School and Hospital of Stomatology Beijing China; ^4^ Department of Clinical Laboratory Union Hospital Tongji Medical College Huazhong University of Science and Technology Wuhan China; ^5^ Department of Respiratory Union Hospital Tongji Medical College Huazhong University of Science and Technology Wuhan China; ^6^ Salivary Gland Disease Center and Molecular Laboratory for Gene Therapy and Tooth Regeneration Beijing Key Laboratory of Tooth Regeneration and Function Reconstruction School of Stomatology Capital Medical University Beijing China

**Keywords:** amblygeustia, COVID‐19 patients, dry mouth, saliva, SARS‐CoV‐2

## Abstract

**Objectives:**

In order to provide a more comprehensive understanding of the effects of SARS‐CoV‐2 on oral health and possible saliva transmission, we performed RNA‐seq profiles analysis from public databases and also a questionnaire survey on oral‐related symptoms of COVID‐19 patients.

**Materials and methods:**

To analyse ACE2 expression in salivary glands, bulk RNA‐seq profiles from four public datasets including 31 COVID‐19 patients were recruited. Saliva and oropharyngeal swabs were collected. SARS‐CoV‐2 nucleic acids in saliva were detected by real‐time polymerase chain reaction (RT‐PCR). Additionally, a questionnaire survey on various oral symptoms such as dry mouth and amblygeustia was also carried out on COVID‐19 patients.

**Results:**

ACE2 expression was present at detectable levels in the salivary glands. In addition, of four cases with positive detection of salivary SARS‐CoV‐2 nucleic acids, three (75%) were critically ill on ventilator support. Furthermore, we observed the two major oral‐related symptoms, dry mouth (46.3%) and amblygeustia (47.2%), were manifested by a relatively high proportion of 108 COVID‐19 patients who accepted the questionnaire survey.

**Conclusions:**

This study confirms the expression of ACE2 in the salivary glands and demonstrates the possibility of SARS‐CoV‐2 infection of salivary glands. Saliva may be a new source of diagnostic specimens for critically ill patients, since it can be easily collected without any invasive procedures. In addition, dry mouth and amblygeustia can be considered as initial symptoms of COVID‐19 infection.

## Graphical abstract

In this study, based on the confirmation of ACE2 receptor expression in salivary glands through database analysis, it was assumed and confirmed that RNA of SARS‐COV‐2 could be detected in saliva samples. At the same time, we also clinically observed that the initial oral‐related symptoms of SARS‐COV‐2 infection may be amblygeustia and dry mouth.

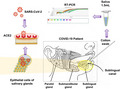

## 1 INTRODUCTION

Since December 2019, an outbreak of a novel coronavirus (SARS‐CoV‐2), which started in Wuhan, China, has been spreading rapidly, resulting in a potentially life‐threatening viral respiratory disease named as coronavirus disease 19 (COVID‐19) by the World Health Organization (WHO).^1^ By 23 August 2020, more than 20 million cases of COVID‐19 and more than 800 000 deaths have been confirmed in over 200 countries, which has been classified as a pandemic by the WHO on 11 March 2020.^2^, ^3^ This in turn had a severely detrimental impact on the public health and economy of several countries worldwide. As one of three diagnostic criteria according to guidelines set by health authorities in China, the positive detection rate of SARS‐CoV‐2 nucleic acids in oropharyngeal swabs of infected patients is relatively high.^4^ At the same time, the presence of SARS‐CoV‐2 in various other human secreta and excreta such as sputum, faeces and urine has also been reported. Nevertheless, as one of the most readily accessible and easily collected bodily fluids, it has not been confirmed whether there are detectable levels of SARS‐CoV‐2 within the saliva of COVID‐19 patients.

Like blood, saliva is rich in multiple biological markers such as DNA, RNA, proteins, with readily detectable levels of microorganism, as the two biofluids share many similarities in molecular components.^5^, ^6^ Hence, there is a high probability of antibodies and viruses of the human body being present in saliva. It has been reported that some viruses of large‐scale infectious diseases, particularly the respiratory diseases such as severe acute respiratory syndrome (SARS) and middle east respiratory syndrome (MERS), can be detected in saliva.^7^, ^8^ Moreover, saliva has already been utilized for the detection of human immunodeficiency virus (HIV), hepatitis B virus (HBV) and various drugs like cocaine and alcohol. ^9^, ^10^ As a result, it is imperative to determine whether SARS‐CoV‐2 can be detected in saliva, as well as characterize the initial oral symptoms manifested in COVID‐19 patients.

Single‐cell RNA‐seq data analysis of angiotensin‐converting enzyme II (ACE2) expression and serological investigation of patient samples have indicated that ACE2 may be the cell receptor of SARS‐CoV‐2,^11^, ^12^ thus suggesting that ACE2‐expressing cells are likely to be the major target cell type which are vulnerable to SARS‐CoV‐2 infection. There is typically high expression of ACE2 r on the epithelial cells of oral mucosa, which is particularly enriched in the epithelial cells of the tongue.^13^ Prior to this study, there have been few reports that specifically investigated whether the salivary gland epithelial cells express ACE2 receptors. To confirm this, bulk RNA‐seq profiles from four public datasets (https://www.proteinatlas.org/) including GTEx dataset, HPA dataset, FANTOM5 dataset and Consensus dataset were analysed. Furthermore, to verify that there are detectable levels of SARS‐CoV‐2 nucleic acids in saliva, we have screened the saliva of COVID‐19 patients (who displayed positive results for SARS‐CoV‐2 nucleic acids detection before or on the day of sample collection). The results have been compared with oropharyngeal swabs, which is one of three diagnostic criteria.^4^ Additionally, we also carried out a survey on the oral health status of COVID‐19 patients, to evaluate the health status of the salivary glands. Our study thus provides a more comprehensive understanding of the detection of SARS‐CoV‐2 in saliva, and initial oral symptoms upon COVID‐19 infection of oral tissues.

## 2 MATERIALS AND METHODS

This study was approved by the Medical Ethics Committee of Union Hospital, Tongji Medical College, Huazhong University of Science and Technology, Wuhan, China (20200062).

### 2.1 Public datasets acquisition

The public bulk RNA‐seq profiles were collected from the Human Protein Altas (available from https://www.proteinatlas.org/) including four datasets (GTEx dataset, HPA dataset, FANTOM5 dataset and Consensus dataset). GTEx data and HPA data were showed as mean pTPM (protein‐coding transcripts per million). FANTOM5 data were obtained through Cap Analysis of Gene Expression (CAGE), which were reported as Scaled Tags Per Million. Data from Consensus were combining the data from GTEx, HPA and FANTOM5 datasets using the internal normalization pipeline, which were reported as Normalized eXpression (NX) levels. Our main focus is on the tissue distribution and expression of ACE2, particularly in the salivary gland.

### 2.2 Inclusion criteria for patients

Inclusion of patients in this study is in accordance with (a) the diagnostic guidelines for new coronavirus pneumonia (NCIP) of the seventh edition,^4^ and (b) SARS‐CoV‐2 nucleic acids detection remaining positive before or on the day of sample collection in our study. The inclusion criteria were set to ensure that the patients we selected in our study were tested positive for SARS‐CoV‐2 nucleic acids before collection of the saliva samples. According to the inclusion criteria described above, 31 patients were selected. According to NCIP of the seventh edition, 26 patients were of the ordinary or heavy type, and 5 patients on ventilator support were of the critically ill type.

### 2.3 Specimen collection

#### 2.3.1 Saliva collection

After cessation of eating or drinking for 30 minutes, the oral cavity of each patient was cleaned by normal saline before saliva collection. Then the tongue of each patient was lifted to expose the opening of the salivary gland duct. Upon gentle massage of the salivary gland, pure saliva was secreted. In this study, we gently collected about 1.5 mL of midstream salivary fluid with cotton swabs. The samples were placed into sterile dry containers immediately, without touching the inside of the cap or inner walls before closing the cap.

#### 2.3.2 Oropharyngeal swab collection

Oropharyngeal swabs and saliva samples were collected at the same time. A synthetic fibre swab was inserted into the patient's throat from the mouth and the posterior pharynx was swabbed, avoiding the tongue. After swabbing, each absorbent swab was placed immediately into a sterile tube.

### 2.4 Nucleic acids extraction and real‐time polymerase chain reaction (RT‐PCR) of SARS‐CoV‐2

According to the latest guidelines issued by WHO, nucleic acids molecular testing of the virus is essential for diagnosis,^14^ with the genetic targets in China being ORF1ab and nCoV‐N.^15^ The SARS‐CoV‐2 was detected by using a RT‐PCR system by following the commercial test kit instructions (BioGerm. InC). The test results were obtained on a Roche Cobas z480 PCR Analyzer. A CT value of less than 35 (or 35 < CT<38 for twice) was defined as positive. Primer sequences used for amplification are listed in Table 1.

**TABLE 1:** Primers used for amplification in detection

ORF1ab	F	CCCTGTGGGTTTTACACTTAA
	R	ACGATTGTGCATCAGCTGA
	P	5′‐FAM‐CCGTCTGCGGTATGTGGAAAGGTTATGG‐BHQ1‐3′
nCoV‐N	F	GGGGAACTTCTCCTGCTAGAAT
	R	CAGACATTTTGCTCTCAAGCTG
	P	5′‐FAM‐TTGCTGCTGCTTGACAGATT‐TAMRA‐3′

### 2.5 Questionnaire for oral symptoms

A questionnaire on the oral health status of patients with infection of SARS‐CoV‐2 was composed and converted into an online format by using the Questionnaire Star website (https://www.wjx.cn/, Questionnaire ID: 60320966) to enquire on the manifestation of 14 oral‐related symptoms by COVID‐19 patients. During the period from 28 February 2020 to 4 March 2020, 108 patients with confirmed infection of SARS‐CoV‐2 were questioned, of which 96 were online and 12 offline, in Wuhan Union Hospital, Wuhan Union Hospital West District and Wuhan Union Hospital Tumor Center. A total of 108 valid questionnaires were collected, of which 52 respondents were male and 56 respondents were female. The online survey data were downloaded from the Questionnaire Star. The SPSS 21.0 software was used to analyse the data, while the GraphPad Prism 7.0 software was used for figure production.

## 3 RESULTS

**FIGURE 1:**
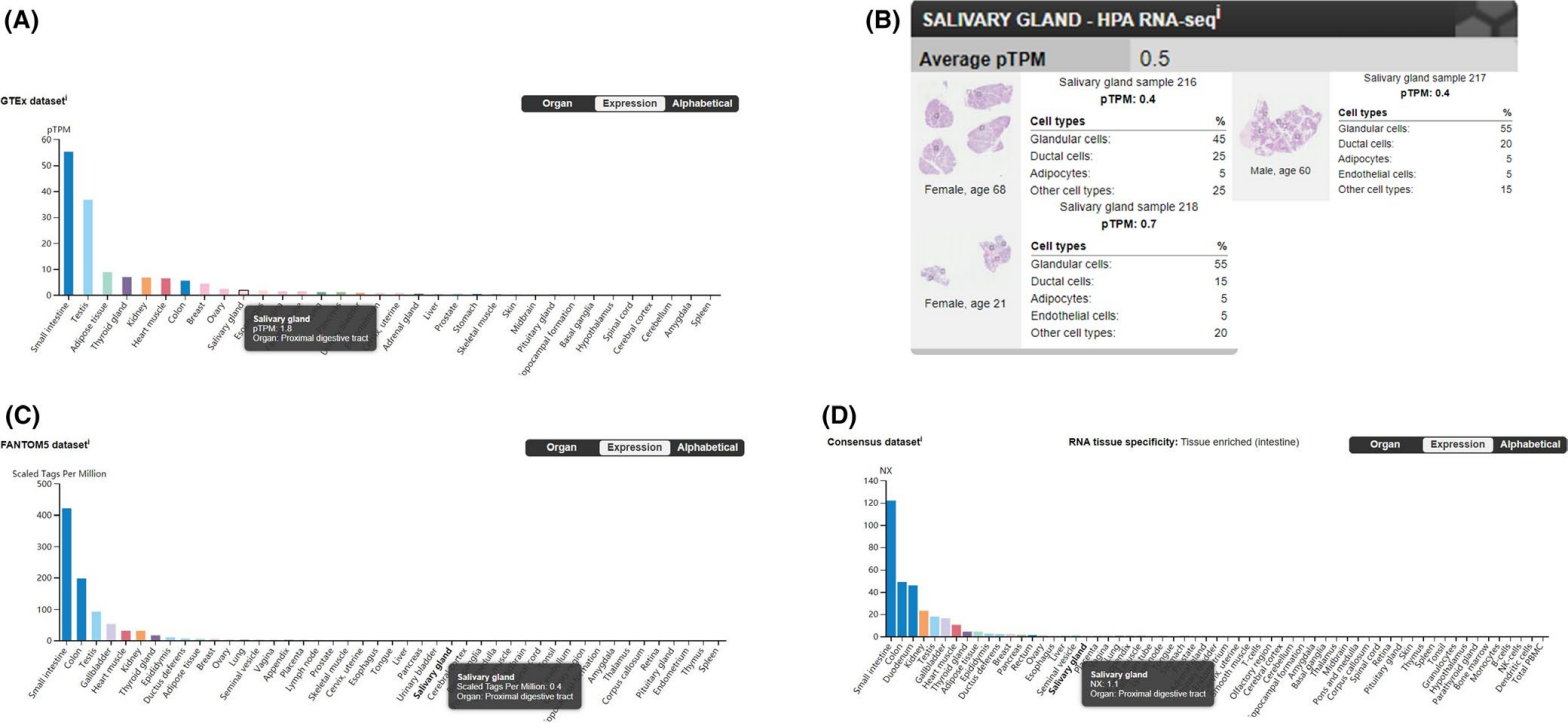
The expression and distribution of ACE2 in normal human tissues. A, Data from GTEx Dataset showing mRNA expression of the ACE2 gene in salivary glands (pTPM: 1.8), with the highest expression level being found in the small intestine (pTPM: 55.2). (Image available from https://www.proteinatlas.org/ENSG00000130234‐ACE2/tissue/). B, Data from HPA Dataset showing mRNA expression of the ACE2 gene being detected in normal salivary gland tissue (average pTPM: 0.5), with the highest level of expression being observed in glandular cells (Image available from https://proteinatlas.org/ENSG00000130234‐ACE2/tissue/Salivary+gland#rnaseq/). C, Data from FANTOM5 Dataset showing mRNA expression of the ACE2 gene in salivary glands (scaled tags per million: 0.4), with the highest expression level being found in the small intestine (scaled tags per million: 420.9). (Image available from https://www.proteinatlas.org/ENSG00000130234‐ACE2/tissue/). D, Data from Consensus Dataset showing mRNA expression of the ACE2 gene by salivary glands (NX: 1.1), with the highest expression level being found in the small intestine (NX: 122.0). (Image available from https://www.proteinatlas.org/ENSG00000130234‐ACE2/tissue/)

The data from GTEx dataset, HPA dataset, FANTOM5 dataset, and Consensus dataset in the Human Protein Atlas (Human Protein Atlas available from https://www.proteinatlas.org/, images/data available from v19.3.proteinatlas.org) showed the expression and distribution of ACE2 within normal human tissues, particularly in salivary glands. The sample ID in GTEx data set, HPA data set and FANTOM5 data set is shown in Tables S1‐S3. The results showed that ACE2 is highly expressed in the gastrointestinal system, testes, kidney and heart muscles. Although ACE2 is also expressed in the salivary glands, its expression is at a relatively low level. The results of RNA‐seq showed that in normal salivary gland tissues, ACE2 is mainly expressed by glandular cells (Figure 1).

In 31 COVID‐19 patients, based on the inclusion criteria, there were 15 male and 16 female patients. The median age was 60.6 years, ranging from 18 to 86 years. There were 5 critically ill patients who needed ventilator support to breathe in these 31 cases. Saliva and oropharyngeal swabs samples were collected at the same time. In our study, 13 cases were tested positive for oropharyngeal swab nucleic acids detection. Among these 13 patients, there were 4 cases with positive nucleic acids detection in saliva, of which 3 cases were critically ill patients on ventilator support, and 1 case being ordinary or heavy without ventilator support (Table 2). The information and test results of the 4 cases are shown in Table 3.

**TABLE 2:** Information and detection results of all included patients

Age (y), N = 31	<30			30~60		>60		
Number	3			10		18		
Gender, N = 31	Male				Female			
Number	15				16			
Main symptoms, N = 31	Cough		Fever		Diarrhoea		Chest tightness	
Number	21		20		4		13	
Type, N = 31	Critically ill				Ordinary or Heavy			
Number	5				26			
Nucleic acids detection results^a^	Negative	Positive			Negative			Positive
Number	1	4			17			9
Nucleic acids detection results in Saliva, N = 13	Negative				Positive			
Number	9				Total			4
					Critically ill			3
					Ordinary or heavy			1

^a^ The results of nucleic acids detection of oropharyngeal swabs.

**TABLE 3:** Information and detection results of critically ill patients with positive detection of nucleic acids in oropharyngeal swabs

Patient number			1	2	3	4
Age (y)			86	66	63	52
Gender			Male	Female	Female	Male
Admission time			02/04	02/14	02/02	02/14
Main symptoms			Cough, fever, weak	Fever, dyspnoea, weak, chest tightness	Cough, fever, dyspnoea	Cough, fever
Time on ventilator			02/17	02/23	02/16	02/18
Detection time (days after using ventilator)			13	7	14	13
Sample type	Oropharyngeal swabs	ORF1ab	+	+	−	+
		nCov‐N	+	+	+	+
	Saliva	ORF1ab	+	−	+	+
		nCov‐N	+	−	+	+

Of the 108 valid questionnaires, 52 respondents were male and 56 respondents were female. The age data of one female patient were missing. After excluding this case, the overall average age was 52.0, 51.1 years for males and 52.9 years for females.

**FIGURE 2:**
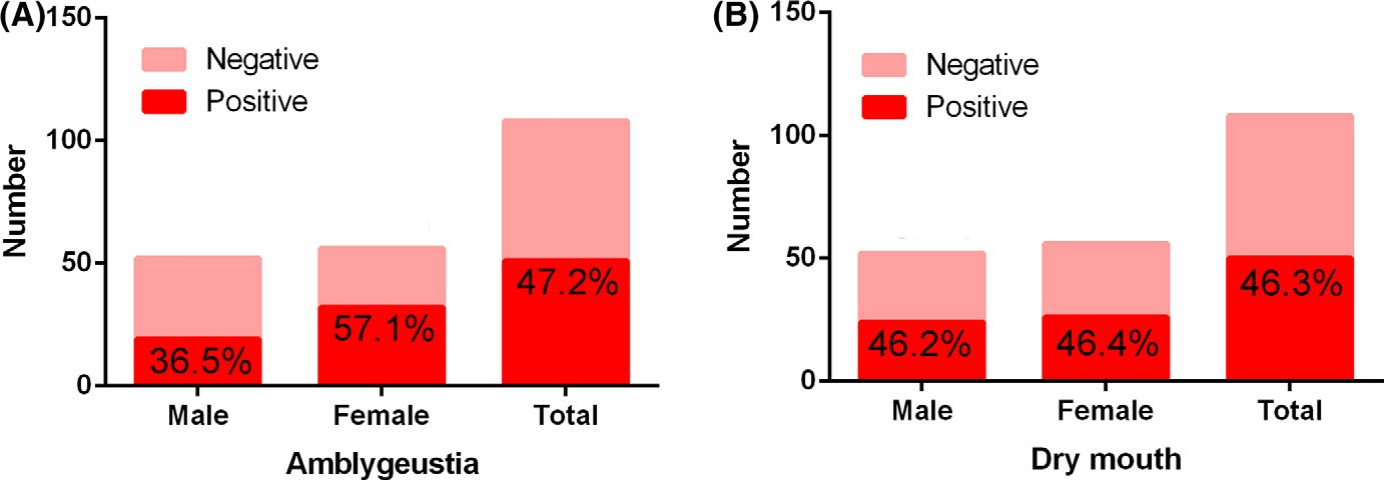
Positive percentages of two oral‐related symptoms with the highest frequency of occurrence during the initial stage of infection. A, Among the oral‐related symptoms listed, amblygeustia positive rate is 47.2% in total, 36.5% in males and 57.1% in females. B, Among the oral‐related symptoms listed, dry mouth positive rate is 46.3% in total, 46.2% in males and 46.4% in females

Among the 14 oral‐related symptoms listed, amblygeustia (47.2% overall, 36.5% in males, 57.1% in females) and dry mouth (46.3% overall, 46.2% in males, 46.4% in females) had the highest incidence. 11.1% (13.5% in males, 8.9% in females) of the patients exhibited dryness and inflammation of mouth. One female patient (0.9% overall, 1.8% of female patients) had enlargement of lymph nodes in the submandibular regions. (Table 4 and Figure 2 present the results of the questionnaire).

**TABLE 4:** Oral‐related symptoms in patients with SARS‐CoV‐2 infection

	Male (N = 52)	Female (N = 55^a^)	Total (N = 107^a^)
Age (M ± SD)	51.1 (15.26)	52.9 (17.28)	52.0 (16.28)
Oral‐related symptoms, n (%)			
	**Male (N = 52)**	**Female (N = 56)**	**Total (N = 108)**
Initial symptoms, n (%)			
Amblygeustia	19 (36.5)	32 (57.1)	51 (47.2)
Dry mouth	24 (46.2)	26 (46.4)	50 (46.3)
Dryness and inflammation of mouth	7 (13.5)	5 (8.9)	12 (11.1)
Enlargement of lymph nodes in the submandibular regions	0	1 (1.8)	1 (0.9)

^a^ Data on the age of 1 female patient were missing.

## 4 DISCUSSION

In our study, we found 4 cases with positive detection of SARS‐CoV‐2 nucleic acids in saliva. This result thus confirmed the possibility of SARS‐CoV‐2 being present in saliva. Hence, the potential transmission risk of salivary fluids should not be ignored. Our discovery is consistent with the tissue distribution of ACE2 expression reported in the public database (https://www.proteinatlas.org/). The ACE2 expression was present at detectable levels in the salivary glands, but was lower than other tissues like the gastrointestinal system, testes, kidney and heart muscles (Figure 1). This might explain why there was just one positive salivary detection in ordinary and heavy patients. A previous study reported the detection rate of SARS‐CoV‐2 in self‐collected saliva.^16^ However, in that study, patients were asked to cough out saliva from their throat into sterile containers, and hence the saliva samples were mainly sputum from the lower respiratory tract. In our study, the saliva samples were the pure fluid from the canal of salivary glands.

It is noteworthy that the positive saliva detection rate was as high as 75% (3/4) in critically ill patients on ventilator support in our study. There might be two possible explanations. First, viral replication in critically ill patients is unchecked by a weakened immune response, so there is a very large amount of virus in the blood or tissue fluids. Hence, the salivary glands are attacked due to the high viral load. Second, in critically ill patients, there is usually a weakened immune system, electrolyte disorders and multiple organ dysfunction, particularly at the late stage of the disease which could destroy the salivary glands and lead to virus invasion of the salivary glands. The results indicated that as SARS‐CoV‐2 infection progressed, the chances of viral particles appearing in the saliva increase. The emergence of the virus in saliva may be an indication that the disease condition of the patient has deteriorated and that the disease has entered the terminal stage. Furthermore, it needs to be emphasized that healthcare workers (HCWs) should pay more attention to increased risks of transmission of COVID‐19 virus through saliva, and that patients with a positive saliva detection may indicate a poor prognostic outcome.

Specifically, for critically ill patients, saliva has a higher potential for detection of SARS‐CoV‐2. Although positive nucleic acids detection of oropharyngeal swab is one of the diagnostic criteria for COVID‐19 testing,^4^ HCWs are exposed to relatively high infection risks while collecting samples. Moreover, collecting oropharyngeal samples may cause discomfort to patients, such as pain, nausea and even bleeding. Therefore, saliva may be a new source of diagnostic specimens for critically ill patients, since it can be easily collected without any invasive procedures, which is advantageous for both HCWs and patients, especially for multiple sampling and continuous monitoring of viral loads.

Another result worth noting in this study is that according to the analysis of the questionnaire, the two major oral‐related symptoms, dry mouth and amblygeustia, were manifested by a relatively high proportion of 108 COVID‐19 patients. A previous study identified fever, fatigue and dry cough as the most common symptoms of COVID‐19 infection.^17^ However, a number of recent studies have found that COVID‐19 patients generally have oral symptoms such as dry mouth and amblygeustia, which are described in a review by Pellegrino et al.^18^ An international multicentre study collected information from 394 patients diagnosed with COVID‐19 from multiple countries, 161 of whom had olfactory or taste disorders, accounting for 41%.^19^ A cross‐sectional study reported that 20% of COVID‐19 patients would feel taste abnormalities.^20^ There have been news reports that even children recovering from mild COVID‐19 are still unable to taste food.^21^ The researchers found a correlation between dry mouth and bitter taste in COVID‐19 patients in a recently published study.^22^ Ren et al also suggest that COVID‐19 patients may develop early oral symptoms (including taste loss and dry mouth), even before fever and dry cough.^23^ Therefore, some experts have suggested that olfactory or taste disorders should be included in COVID‐19 screening criteria.^24^ Oral health researchers may play a more active role in the early diagnosis and treatment of COVID‐19 by exploring the mechanism of dry mouth and amblygeustia.

Besides viral invasion, we speculate that oral symptoms may also be due to the patient's change in psychological status, poor oral hygiene or microbiota imbalance caused by therapeutic drugs. In particular, high ACE2 expression can be found on the epithelial cells of oral mucosa, which is enriched in epithelial cells of the tongue, thus providing possible routes of entry for SARS‐CoV‐2.^13^ Therefore, oral mucosa might be at potential risk of infection by SARS‐CoV‐2, suggesting that oral symptoms could also be considered as initial symptoms of COVID‐19 infection, which might be new diagnostic criteria. At the same time, doctors should pay more attention to the oral condition and oral hygiene of patients, and give proper oral treatment during the clinical treatment process.

This study thus confirms that ACE2 is also expressed in the salivary glands, SARS‐CoV‐2 can be detected in saliva, and oral symptoms may be frequently manifested by COVID‐19 patients. The findings of this study suggest that saliva may carry a risk of SARS‐CoV‐2 transmission, particularly in critically ill patients, and that SARS‐CoV‐2 could cause partial impairment of oral tissues, thus providing a new insight to the clinical prevention, diagnosis and treatment of COVID‐19. More in vitro and in vivo evidence and in‐depth histological data are needed to further confirm and reinforce these findings.

## CONFLICT OF INTEREST

We declare no competing interests.

## AUTHOR CONTRIBUTIONS

S. W., L. W. and Y. J. designed and supervised this study, and revised the manuscript; L. C. carried out the experiments, analysed the data, wrote and revised the article; J. Z. and J. P. searched the databases, analysed the data, wrote and revised the article; X. D. analysed the data and revised the article; X. L., Z. S. and F. G. collected clinical samples and case data; Z. G. and Q. Z. performed the laboratory testing.

## Supporting information

Table S1‐S3

## Data Availability

The data that support the findings of this study are available on request from the corresponding author.
