# Supplementary material for: Detection of SARS‐CoV‐2 in saliva and characterization of oral symptoms in COVID‐19 patients
Source: Cell Prolif. 2020 Oct 19;53(12):e12923. doi: 10.1111/cpr.12923 (PMC7645955; doi:10.1111/cpr.12923)
Supplement: Supplementary file 1 — Table S1‐S3 [file CPR-53-e12923-s001.docx]

**Supplementary materials**

**Table S1. The sample ID of salivary glands in GTEx dataset**

| GTEx sample ID | Sample description | pTPM |
| --- | --- | --- |
| GTEX-18D9U-2226-SM-7KFTN | 40-49 years, male | 7.2 |
| GTEX-13OVK-1826-SM-6PALE | 40-49 years, male | 5.6 |
| GTEX-12WSL-2126-SM-5GCNG | 50-59 years, male | 4.9 |
| GTEX-1AX9K-2126-SM-72D7K | 50-59 years, male | 4.3 |
| GTEX-1339X-2326-SM-5HL5B | 40-49 years, male | 4.3 |
| GTEX-11P82-0926-SM-5986X | 20-29 years, male | 4.2 |
| GTEX-YF7O-2226-SM-5CVN8 | 50-59 years, male | 3.6 |
| GTEX-1C64O-0626-SM-7DHL1 | 60-69 years, male | 3.5 |
| GTEX-1GPI7-0326-SM-7MKH6 | 20-29 years, male | 3.4 |
| GTEX-15EU6-2626-SM-7KUN4 | 50-59 years, male | 3.3 |
| GTEX-14PJ3-1526-SM-664O9 | 50-59 years, male | 3.2 |
| GTEX-ZA64-1726-SM-5CVMF | 20-29 years, male | 3.2 |
| GTEX-YB5K-1726-SM-5IFIZ | 40-49 years, female | 3.2 |
| GTEX-178AV-1426-SM-6LLID | 40-49 years, male | 3.2 |
| GTEX-13OVI-2326-SM-5KM47 | 60-69 years, female | 3.1 |
| GTEX-11P81-2026-SM-5P9GT | 30-39 years, female | 3 |
| GTEX-1B97J-2126-SM-79OL6 | 50-59 years, female | 3 |
| GTEX-15RJE-2726-SM-6LPJ1 | 40-49 years, male | 2.9 |
| GTEX-13QJC-2326-SM-5Q5CD | 60-69 years, female | 2.9 |
| GTEX-WH7G-2126-SM-4M1XU | 40-49 years, male | 2.9 |
| GTEX-1314G-2026-SM-5LZUM | 30-39 years, male | 2.8 |
| GTEX-Y3I4-1926-SM-4TT8L | 50-59 years, male | 2.7 |
| GTEX-ZP4G-1926-SM-57WDZ | 20-29 years, female | 2.7 |
| GTEX-1AX8Z-0626-SM-731EH | 60-69 years, male | 2.5 |
| GTEX-ZF29-2126-SM-4WWB8 | 60-69 years, female | 2.5 |
| GTEX-WHSB-2326-SM-4M1XK | 50-59 years, male | 2.5 |
| GTEX-13O21-1926-SM-5MR39 | 50-59 years, male | 2.2 |
| GTEX-WY7C-1426-SM-4OND1 | 50-59 years, male | 2.2 |
| GTEX-WFJO-2026-SM-4LVM3 | 30-39 years, male | 2.2 |
| GTEX-1AMEY-1126-SM-72D79 | 30-39 years, female | 2 |
| GTEX-145ME-1626-SM-5RQI3 | 40-49 years, female | 2 |
| GTEX-XGQ4-2626-SM-4AT6B | 50-59 years, male | 1.9 |
| GTEX-11EQ9-2026-SM-5P9JI | 30-39 years, male | 1.9 |
| GTEX-WYVS-2026-SM-4RGNS | 40-49 years, female | 1.9 |
| GTEX-18QFQ-0626-SM-718AW | 30-39 years, male | 1.8 |
| GTEX-1EH9U-2926-SM-7DHLX | 60-69 years, male | 1.8 |
| GTEX-ZQG8-2126-SM-4YCF1 | 60-69 years, female | 1.8 |
| GTEX-15CHC-1226-SM-686ZH | 60-69 years, female | 1.7 |
| GTEX-18A7B-2826-SM-7KFTK | 70-79 years, male | 1.7 |
| GTEX-X15G-2126-SM-4PQZR | 50-59 years, female | 1.7 |
| GTEX-X261-2426-SM-4PQZU | 50-59 years, male | 1.7 |
| GTEX-18A67-0626-SM-7KFSL | 50-59 years, male | 1.6 |
| GTEX-ZT9W-2126-SM-51MSF | 50-59 years, male | 1.6 |
| GTEX-12BJ1-2226-SM-5BC6G | 60-69 years, male | 1.6 |
| GTEX-12WSN-2226-SM-5DUXT | 40-49 years, male | 1.6 |
| GTEX-1CB4I-2526-SM-7MKG1 | 50-59 years, male | 1.6 |
| GTEX-13111-2326-SM-5LZUQ | 50-59 years, male | 1.6 |
| GTEX-XMK1-2626-SM-4B65R | 40-49 years, male | 1.6 |
| GTEX-Y5V6-2226-SM-4VDTC | 60-69 years, male | 1.5 |
| GTEX-1117F-3026-SM-5GZYU | 60-69 years, female | 1.5 |
| GTEX-1GMR2-2026-SM-7MKHK | 50-59 years, male | 1.5 |
| GTEX-146FR-1226-SM-5SIA7 | 60-69 years, female | 1.5 |
| GTEX-WOFM-2126-SM-4OOSR | 20-29 years, male | 1.4 |
| GTEX-1B932-0626-SM-731EM | 40-49 years, female | 1.3 |
| GTEX-13QBU-2126-SM-5KLZN | 40-49 years, female | 1.3 |
| GTEX-ZPU1-2326-SM-57WEP | 40-49 years, male | 1.3 |
| GTEX-14PJN-1726-SM-69LO7 | 30-39 years, male | 1.3 |
| GTEX-Y5LM-1826-SM-4VDT9 | 40-49 years, female | 1.2 |
| GTEX-X5EB-1726-SM-4E3J7 | 40-49 years, male | 1.2 |
| GTEX-ZXG5-2826-SM-5NQ8T | 60-69 years, male | 1.2 |
| GTEX-17KNJ-2726-SM-7KFRE | 50-59 years, male | 1.2 |
| GTEX-13JVG-2926-SM-5J2N9 | 60-69 years, male | 1.2 |
| GTEX-13VXT-1726-SM-5IFF5 | 20-29 years, female | 1.2 |
| GTEX-13113-1526-SM-5EGHV | 60-69 years, female | 1.2 |
| GTEX-17MFQ-1626-SM-7938Z | 20-29 years, male | 1.1 |
| GTEX-1A3MV-1726-SM-72D68 | 30-39 years, male | 1.1 |
| GTEX-11ZTS-3026-SM-5EQM7 | 60-69 years, female | 1.1 |
| GTEX-14B4R-1426-SM-5Q5CG | 30-39 years, male | 1 |
| GTEX-XV7Q-2426-SM-4BRV8 | 40-49 years, female | 1 |
| GTEX-X3Y1-1926-SM-4PQZO | 40-49 years, male | 1 |
| GTEX-131XF-2826-SM-5EGKH | 60-69 years, male | 1 |
| GTEX-14E1K-2626-SM-664OJ | 50-59 years, male | 1 |
| GTEX-1192X-2926-SM-5N9BA | 50-59 years, male | 1 |
| GTEX-15SHV-2226-SM-6M464 | 60-69 years, male | 0.9 |
| GTEX-12WSM-1826-SM-5BC64 | 60-69 years, male | 0.9 |
| GTEX-131XH-2626-SM-5GCNP | 50-59 years, male | 0.9 |
| GTEX-11EMC-2526-SM-5PNVU | 60-69 years, female | 0.8 |
| GTEX-11WQK-2926-SM-5EQKT | 50-59 years, male | 0.8 |
| GTEX-1C64N-2926-SM-79OLM | 50-59 years, male | 0.8 |
| GTEX-14PN4-2526-SM-686ZG | 50-59 years, male | 0.7 |
| GTEX-1B8KZ-1726-SM-73KV7 | 50-59 years, male | 0.7 |
| GTEX-1C4CL-2026-SM-731EB | 30-39 years, male | 0.6 |
| GTEX-11ZTT-2226-SM-5CVLP | 60-69 years, female | 0.6 |
| GTEX-1445S-2926-SM-5O993 | 60-69 years, male | 0.6 |
| GTEX-13G51-2426-SM-5LZY8 | 60-69 years, male | 0.5 |
| GTEX-13OVL-2926-SM-5IFG3 | 50-59 years, male | 0.5 |
| GTEX-Y111-2626-SM-4TT24 | 50-59 years, male | 0.4 |
| GTEX-11NV4-1926-SM-5N9D4 | 60-69 years, male | 0.4 |
| GTEX-ZQUD-2126-SM-51MSY | 30-39 years, male | 0.4 |
| GTEX-1B8SF-1826-SM-731DJ | 60-69 years, male | 0.4 |
| GTEX-13CF2-2426-SM-5LZYL | 60-69 years, male | 0.3 |
| GTEX-ZC5H-2726-SM-5KLZW | 40-49 years, female | 0.3 |
| GTEX-111FC-2926-SM-5GZY7 | 60-69 years, male | 0.3 |
| GTEX-13NYB-2426-SM-5IFF4 | 40-49 years, male | 0.2 |
| GTEX-ZE7O-2926-SM-51MSG | 50-59 years, female | 0.2 |
| GTEX-11TUW-2426-SM-5EQKB | 60-69 years, male | 0.2 |
| GTEX-14JIY-3026-SM-6EU1R | 60-69 years, male | 0.2 |

**Table S2. The sample ID of salivary glands in HPA dataset**

| **HPA sample ID** | **Sample description** | **pTPM** |
| --- | --- | --- |
| Salivary gland sample 216 | Female, age 68 | 0.4 |
| Salivary gland sample 217 | Male, age 60 | 0.4 |
| Salivary gland sample 218 | Female, age 21 | 0.7 |

**Table S3. The sample ID of salivary glands in FANTOM5 dataset**

| **FANTOM5 sample ID** | **Sample description** | **Scaled Tags Per Million** |
| --- | --- | --- |
| FF:10199-103F1 | 23 years, male | 0.4 |
| FF:10093-102C3 | 16-60 years, mixed | 0.1 |
| FF:10202-103F4 | 24 years, male | 0.4 |

*Data of Table S1, Table S2 and Table S3 available from https://www.proteinatlas.org/ENSG00000130234-ACE2/tissue/Salivary+gland#rnaseq/
